# Role of vimentin in modulating immune cell apoptosis and inflammatory responses in sepsis

**DOI:** 10.1038/s41598-019-42287-7

**Published:** 2019-04-05

**Authors:** Longxiang Su, Pan Pan, Peng Yan, Yun Long, Xiang Zhou, Xiaoting Wang, Ruo Zhou, Bo Wen, Lixin Xie, Dawei Liu

**Affiliations:** 1 Department of Critical Care Medicine, Peking Union Medical College Hospital, Peking Union Medical College, Chinese Academy of Medical Sciences, 1 Shuaifuyuan, Dongcheng District, Beijing, 100730 China; 2 Department of Critical Care Medicine, Beijing Tiantan Hospital, Capital Medical University, Tiantan Xili the 6th, Dongcheng District, Beijing, 100050 China; 3 Department of Respiratory and Critical Care Medicine, Chinese PLA General Hospital, 28th Fuxing Rd, Haidian District, Beijing, 100853 China; 4 Shenzhen Proteome Engineering Laboratory, BGI Shenzhen, Shenzhen, 518083 Guangdong Province China

## Abstract

New diagnostic biomarkers or therapeutic targets for sepsis have substantial significance for critical care medicine. In this study, 192 differentially expressed proteins were selected through iTRAQ. Based on cluster analysis of protein expression dynamics and protein-protein interactions, hemopexin, vimentin, and heat shock protein 90 were selected for further investigation. It was demonstrated that serum vimentin (VIM) levels were significantly increased in patients with sepsis and septic shock compared to controls and that VIM expression was significantly increased in lymphocytes isolated from septic shock and sepsis patients compared to controls. Moreover, a nonsurvivor group had higher serum VIM levels and VIM expression in lymphocytes. Caspase-3 was significantly upregulated in Jurkat T cells lacking VIM and when exposed to LPS compared to control cells. In contrast, caspase-3 was reduced nearly 40% in cells over-expressing VIM. IL-2, IL-10 and IFN-α levels were significantly decreased in cells lacking VIM compared to control cells, whereas they were not significantly altered in cells over-expressing VIM. These findings suggest that VIM modulates lymphocyte apoptosis and inflammatory responses and that VIM could be a new target for the diagnosis and prognostic prediction of patients with sepsis or septic shock.

## Introduction

Sepsis refers to the presence of a serious infection in combination with organ dysfunction^1^. Due to sepsis’s significant clinical heterogeneity and nonspecific characteristics, timely and accurate diagnosis of sepsis—as well as prognosis of disease severity—is clinically difficult. Although significant advances in the clinical treatment of sepsis have been made, the mortality associated with sepsis remains high^2^. It has been reported that nearly 250,000 cases of sepsis result in death in the USA annually^3^, equivalent to approximately 59.6 deaths per 100,000 individuals^4^. In China, it has been reported that nearly 37 of every 100 ICU admissions were for severe sepsis or septic shock, and the overall ICU and hospital mortality rates were 28.7% and 33.5%, respectively^5^. The development of sepsis is an extremely complex and rapid pathophysiological process involving inflammation/anti-inflammation cascades, humoral/cellular immunity, and hemodynamic abnormalities^6^.

Once a diagnosis of sepsis is made, and standard treatment initiated, it is often difficult to differentiate between patients more likely to achieve positive, rather than negative, outcomes. Biomarkers have the potential to guide clinicians to extend treatment beyond standard regiments and to predict patient outcomes. Two types of biomarkers can be identified in clinical practice: diagnostic or prognostic biomarkers used independent of therapy and biomarkers used as an adjunct to guide treatment^7^. Regardless of classification, the demand for new and accurate biomarkers for sepsis or septic shock is high. In 2010, Pierrakos and Vincent estimated that at least 178 different sepsis biomarkers have been reported in the scholarly literature^8^; 4 years later, Sandquist and Wong reported that soluble triggering receptor expressed on myeloid cells-1 (sTREM-1), IL-27, soluble urokinase-type plasminogen activator receptor (suPAR), neutrophil CD64, presepsin, cell-free DNA (cfDNA), and certain miRNAs could be potential biomarkers for the diagnosis, prognosis and treatment of sepsis^9^.

With recent developments and increased use of basic biotechnological methods, including genomics, transcriptomics, proteomics and metabolomics platform technologies in biomedical and clinical research, newly discovered potential biomarkers for sepsis diagnosis and prognosis have increased. In this context, we have focused on the application of clinical proteomics and metabolomics for the diagnosis and prognosis of sepsis. Specifically, we have assessed various urinary biomarkers at different stages of sepsis development and identified lysosome-associated membrane protein-1 (LAMP-1) as being closely related to a poorer prognosis, which revealed the important role of autophagy during the progression of sepsis^10–12^. Additionally, we have also previously reported the important role of sulfur-containing amino acids and vitamin D in sepsis^11,13^. We have further performed proteomics analysis of blood samples collected from patients with sepsis or septic shock, and we found that serum vimentin levels were significantly increased in these patients.

Vimentin is an intermediate filament expressed in variety of cells, including lymphocytes and neutrophils. Recent studies have suggested that vimentin plays a role in many cell and tissue functions, including apoptosis of neutrophils and lymphocytes^14–18^. Since apoptosis of innate immune cells—particularly lymphocyte apoptosis—has been recognized as an important step in the pathogenesis of sepsis, and as vimentin plays a role in lymphocyte apoptosis, we hypothesized that disruption of vimentin in lymphocytes results in cell death and that soluble vimentin released by apoptotic cells into blood circulation could serve as a potential biomarker for the prognosis of sepsis. To test this hypothesis, the current study was carried out in the following three stages: screening target proteins from patient blood samples and selecting vimentin as a target protein, validation of the vimentin in the clinical setting of sepsis, and *in vitro* experimental studies to explore the role of vimentin in regulating apoptosis of lymphocytes in response to endotoxin exposure.

## Materials and Methods

### Patients

Patients who were admitted to the Respiratory Intensive Care Unit (ICU), Surgical ICU, and Emergency ICU at Chinese People’s Liberation Army (PLA) General Hospital from May 2010 to Oct 2011 were enrolled in the current study. Systemic Inflammatory Response Syndrome (SIRS) and sepsis were diagnosed based on the 1991 ACCP/SCCM Sepsis Directory^19^ and the revised criteria in the 2001 and 2008 updates^20,21^. Patients with two or more of the following signs were eligible for SIRS diagnosis: (1) a temperature >38 °C or <36 °C, (2) a pulse rate >90 beats/min, (3) a respiratory rate >20 breaths/min or hyperventilation with a partial pressure of arterial carbon dioxide (PaCO_2_) <32 mmHg, or (4) a white blood cell (WBC) count >12,000 µL^−1^ or <4,000 µL^−1^, or >10% immature cells. Severe sepsis was defined as sepsis combined with organ dysfunction. Once a vasoactive drug had to be used after fluid resuscitation, the case was defined as septic shock. In this study, 100 participants were randomly enrolled and assigned to a SIRS (n = 20), sepsis (n = 20), severe sepsis (consisting of severe sepsis, n = 10; and septic shock, n = 10), or death groups (patients with septic shock who died during treatment, n = 20). For the control group, 20 healthy volunteers were enrolled. The study protocol of this stage was approved by the Ethics Committee of the Chinese PLA General Hospital (project No. 20111013-007) and was registered with the Clinical Trials Register (NCT01493466).

The clinical validation stage utilized a prospective study conducted from July 2016 to December 2016 in the Department of Critical Care Medicine, Peking Union Medical College Hospital. Sepsis 3.0 was adopted to select patients for validation of potential proteins^1^. Sepsis was then defined as life-threatening organ dysfunction (an acute change in total SOFA score ≥2 points) caused by a dysregulated host response to infection. Patients with septic shock were identified based on a clinical presentation of sepsis with persistent hypotension requiring vasopressors to maintain a MAP ≥65 mmHg and a serum lactate level >2 mmol/L despite adequate volume resuscitation. A total of 31 patients were randomly selected and enrolled in this stage of study, and these patients were divided into sepsis (n = 12) and septic shock (n = 19) groups. On day 28, these patients were further classified as survivors (n = 21) and nonsurvivors (n = 10). Additionally, 10 surgical postoperative, noninfected patients were assigned to the control group. The study protocol of this stage was approved by the Ethics Committee of the Peking Union Medical College Hospital (project No. ZS1080) and was registered with the Clinical Trials Register (NCT 03253146).

Patients were excluded if they were (1) younger than 18 years; (2) had acquired immunodeficiency syndrome; (3) had reduced polymorph nuclear granulocyte counts (<500 μL^−1^); (4) died within 24 h after admission to the ICU; (5) refused to participate in the study; or (6) declined treatment during the period of observation. Participants or their family members were fully informed regarding the study and then provided signed informed consent before participation in the study. All experiments were performed in accordance with relevant guidelines and regulations.

### Sample Collection

In the clinical screening stage, blood samples were collected from patients within 24 h of admission to the ICU. For the iTRAQ proteomic assay (Applied Biosystems), serum proteins from patients within the same stages/groups and the controls were pooled into one subgroup to minimize individual variation and enhance signals^22^. Five independent sample subgroups, control (CON), SIRS (SI), sepsis (SE), severe sepsis (SS), and the death (DE), were used for screening by iTRAQ labeling. The blood specimens for the clinical validation were also collected from an additional 41 participants within 24 h after ICU admission, and these blood specimens were further separated into two parts that were used for ELISA quantification of the serum proteins and flow cytometry-based evaluation of the blood cells.

### iTRAQ Labeling and LC-ESI-MS/MS Analysis

Proteins were concentrated with a commercial kit (ProteoMiner^TM^ Protein Enrichment Large-Capacity Kit, BIO-RAD, cat# 163-3007) and quantified with a commercial assay kit (Bradford Protein Assay Kit, CWBIO, cat# CW0013). Trypsin Gold (Promega, Madison, WI, USA) was then used to digest the samples into peptides. Peptides were reconstituted in 0.5 M TEAB and processed following the manufacturer’s protocol for 8-plex iTRAQ (Applied Biosystems). Further details of the protocol used for protein mass spectrometry can be found in our previously published study^10^. The experiment was repeated three times.

### Database Search and Bioinformatics analysis

The International Protein Index (IPI) human sequence databases (version 3.83, HUMAN, 93,289 sequences) were used to search the MS/MS spectra with MASCOT software (Matrix Science, London, U.K.; version 2.2). All identified peptides had an ion score above the Mascot peptide identity threshold, and a protein was considered successfully identified if at least one such unique peptide match was apparent for the protein. The protein-abundance ratios were set at a 1.2-fold threshold change, and a two-tailed *p*-value of <0.05 was used to identify significant changes.

The differentially expressed proteins were detected as part of a logical sequence according to random variance model corrective ANOVA. In accordance with different signal density change tendencies of proteins under different situations, a set of unique model expression tendencies was identified. The raw expression values were converted to log2 ratios. Using a strategy for clustering time-series protein expression data, we defined certain unique profiles. The expression model profiles were related to the actual or the expected number of proteins assigned to each model profile. Significant profiles had a higher probability than expected by Fisher’s exact test and multiple comparison tests^23,24^.

To investigate the global network, we computationally identified the most important nodes based on the source of the interaction database from KEGG. To this **e**nd, we turned to the connectivity (also known as degree), defined as the sum of connection strengths with the other network genes:${{\rm{K}}}_{i}=\sum _{u\ne i}{a}_{ui}$. In protein networks, the connectivity measures how correlated a protein is with all its other network proteins. For a protein in the network, the number of source proteins of a protein is referred to as the in-degree of the protein, and the number of target proteins of a protein is referred to as the out-degree. The character of proteins is described by betweenness centrality measures reflecting the importance of a node in a graph relative to other nodes. For a given graph G:(V, E) with n vertices, the relative betweenness centrality ${C^{\prime} }_{B}(v)$ is defined as $${C^{\prime} }_{B}(v)=\frac{2}{{n}^{2}-3n+2}\sum _{\begin{array}{c}s\ne v\ne t\in V\\ s\ne t\end{array}}\frac{{\sigma }_{st}(v)}{{\sigma }_{st}}$$, where ${\sigma }_{st}$ is the number of shortest paths from s to t, and ${\sigma }_{st}(v)$ is the number of shortest paths from s to t that pass through a vertex v^25–29^. The graph nature of the networks was drawn with powerful tools implemented in R software (http://www.r-project.org) and registered in the State Intellectual Property Office of the People’s Republic of China (No. CN102289432A; http://epub.sipo.gov.cn/).

### Cell culture and treatment

Jurkat T cells (CRL-2898) were purchased from American Type Collection of Culture (Manassas, VA, USA) and cultured in DMEM supplemented with 10% fetal calf serum (FCS), penicillin/streptomycin and amphotericin B. Cells were split every 5–7 days. For experiments, cells were treated with or without 10 µg/ml lipopolysaccharides (LPS) as indicated below.

### Transfection of siRNA and plasmids

For siRNA transfection, cells were plated in 60 mm dishes (3 × 10^6^ cells/dish) in 1 mL Opti-MEM. Negative control siRNA (Santa Cruz Biotechnology, Cat#: sc-37007) or vimentin-specific siRNA (Santa Cruz Biotechnology, Cat#: sc-29522) were mixed with Lipofectamine 2000 (Santa Cruz Biotechnology, Cat#: sc-29528) following the manufacturer’s instruction in Opti-MEM. The mixture was then added to the cells (500 µL/dish, final concentration of siRNA was 200 nM in 1.5 mL media/dish). After 6 h of transfection, cells were further cultured for 24 h with 10% FCS-supplemented DMEM containing antibiotics and amphotericin B overnight. Cells were then used for experiments as described.

For transfection of the vimentin-overexpressing plasmid, cells were plated in 60 mm dishes (3 × 10^6^ cells/dish) in 1 mL Opti-MEM. The vimentin-expressing plasmid (pCMV3-VIM, SinoBiological Inc., Beijing, China) or a negative control (pCMV3-untagged vector, SinoBiological Inc., Beijing, China) was mixed with transfection reagent (Sinofcetion-293, Sino Biological Inc., Beijing, China) following the manufacturer’s instructions in Opti-MEM. The mixture was then added to the cells (500 µL/dish, final concentration of siRNA was 200 nM in 1.5 mL media/dish). After 6 h of transfection, cells were further cultured for 24 h with 10% FCS-supplemented DMEM containing antibiotics and amphotericin B overnight. Cells were then used for experiments as described.

### TUNEL Assay

Apoptosis was assessed using a TUNEL assay kit (Roche Molecular Biochemicals) following the manufacturer’s instruction. Briefly, after the desired treatment, the cells were spun on a slide (100 µL/slide) with a cytospin device (Cytospin 2, Shandon, USA) at 1000 rpm for 5 min. After air-drying and fixation with 10% formalin, the cells were permeabilized with 0.1% Triton X-100 in 0.1% sodium citrate for 2 min at 4 °C. The cells were then incubated with the TUNEL mixture in a humidified chamber for 60 min at 37 °C in the dark. After counterstaining with 1 µg/mL of propidium iodide, the fluorescence incorporation into nuclei was detected and imaged under a fluorescence microscope at 200x magnification (Leica, Germany). TUNEL-positive and total cell numbers were counted in at least 5 random fields for each slide by two independent researchers, and data are presented as the mean ± SEM of the numbers counted by the two researchers.

### Immunoblotting

Cells were lysed with RIPA buffer (50 mM Tris-HCL, pH 7.4, 50 mM NaCl, 2 mM EDTA, 0.1% SDS plus freshly added proteinase inhibitor cocktail (including apoprotein, leupeptin, DTT and PMSF)). Protein concentrations in the supernatant were determined using a protein dye-binding assay (Bio-Rad). Total proteins were then subjected to immunoblot analysis. Briefly, after heating for 5 min at 95 °C followed by cooling on ice, 10 µg of total protein was mixed with 2X sample loading buffer (0.5 M Tris-HCL, pH 6.8, 10% SDS, 0.1% bromophenol blue, 20% glycerol, 2% ß-mercaptoethanol) and loaded into each lane before performing electrophoresis with a Mini-protein 3 Cell System (Bio-Rad). The proteins were transferred to a PVDF membrane (Bio-Rad) in transfer buffer (20 mM Tris, pH 8.0, 150 mM glycine, 20% methanol) with a semi-dry electrophoretic transfer system (Bio-Rad). The membrane was then blocked with blocking buffer (Li-COR, Lincoln, USA) at room temperature for 1 h, and then exposed to primary antibodies (Santa Cruz Biotechnology) at 4 °C overnight. Targeted proteins were subsequently detected using IRDye 800CW goat anti-mouse antibody (Li-COR, Lincoln, USA) for 1 h at room temperature and dark. Bands were visualized with an image scanner (Li-COR, Lincoln, USA).

### Cytokine quantification by ELISA

Concentrations of cytokines in the supernatants of cultures were quantified using a DuoSet ELISA kit (R&D System, Minneapolis, MN, USA) following the manufacturer’s instructions with slight modification. Briefly, 96-well plates were coated with monoclonal antibodies at 4 °C overnight. Standards or samples were applied to individual wells and incubated at 4 °C overnight followed by washing and the application of biotinylated antibodies for 1 h at room temperature. After washing, HRP–streptavidin conjugate was added for 1 h at room temperature followed by detection with TMB (Sigma). The reaction was stopped with 1 M H_2_SO_4_ and quantified at 450 nm with a microplate reader (Bio-Rad, Hercules, CA, USA).The limits of detectability for human cytokine assays were as follows: IL-1ß, 3.91 pg/mL; IL-2, 25 pg/mL; IL-10, 31.2 pg/mL; IL-12, 31.2 pg/mL; TNF-γ, 15 pg/mL, and IFN-α, 7.8 pg/mL. Serum VIM levels wee examined by a double antibody sandwich ELISA (Human Vimentin (VIM) ELISA Kit, CUSABIO, Catalog Number. CSB-E08982h). ELISA was performed in duplicate, and other assays were performed in strict accordance with the manufacturers’ instructions.

### Flow Cytometry

Within 3 h of the collection of peripheral arterial blood, a 500-μl aliquot of each sample was taken. The 500-μl aliquots were added to red blood cell lysis solution (QBLysing Solution, QuanBio, Beijing, China) and diluted to 1 × 10^6^ cells/ml. Cells were then incubated with Flow Cytometry Fixation/Permeabilization Buffer I (R&D Systems, Catalog # FC007) at 4 °C for 30 min. To maintain a single-cell suspension, the cells were briefly vortexed followed by centrifugation. The cell pellet was resuspended in 200 μL of the Flow Cytometry Permeabilization/Wash Buffer I (R&D Systems, Catalog # FC005). FITC-conjugated mouse anti-human-vimentin (ab128507, Abcam, Cambridge, MA) was added to the cells at the optimal concentration. The mixture was incubated for 30 min at 4 °C. Excess antibody was removed by washing the cells once in 1 mL of Flow Cytometry Permeabilization/Wash Buffer I. The cell pellet was resuspended in 200 μL of Flow Cytometry Staining Buffer (R&D Systems, Catalog # FC001) for flow cytometric analysis. The fluorescence intensities were measured by flow cytometry (BD Accuri™ C6 Plus flow cytometer, Accuri Cytometers Inc., Cambridge, UK) and analyzed with CFLow Plus Software (Accuri Cytometers Inc., Cambridge, UK). Fresh peripheral blood cells were sorted into monocytes, lymphocytes and neutrophils using FCM.

### Statistical Analysis

Continuous variables with or without normal distributions are presented as means ± standard deviations (SD) and medians (interquartile range), respectively. One-way ANOVA or Mann-Whitney U tests were used to compare means between the different groups. Chi-square testing was performed to compare between groups with qualitative variable data. Statistical analyses were conducted with SPSS 16.0 (SPSS, Chicago, IL, USA), and a two-tailed *p-value* < 0.05 was considered to be significant.

## Results

### Demographics of subjects

**Figure 1:**
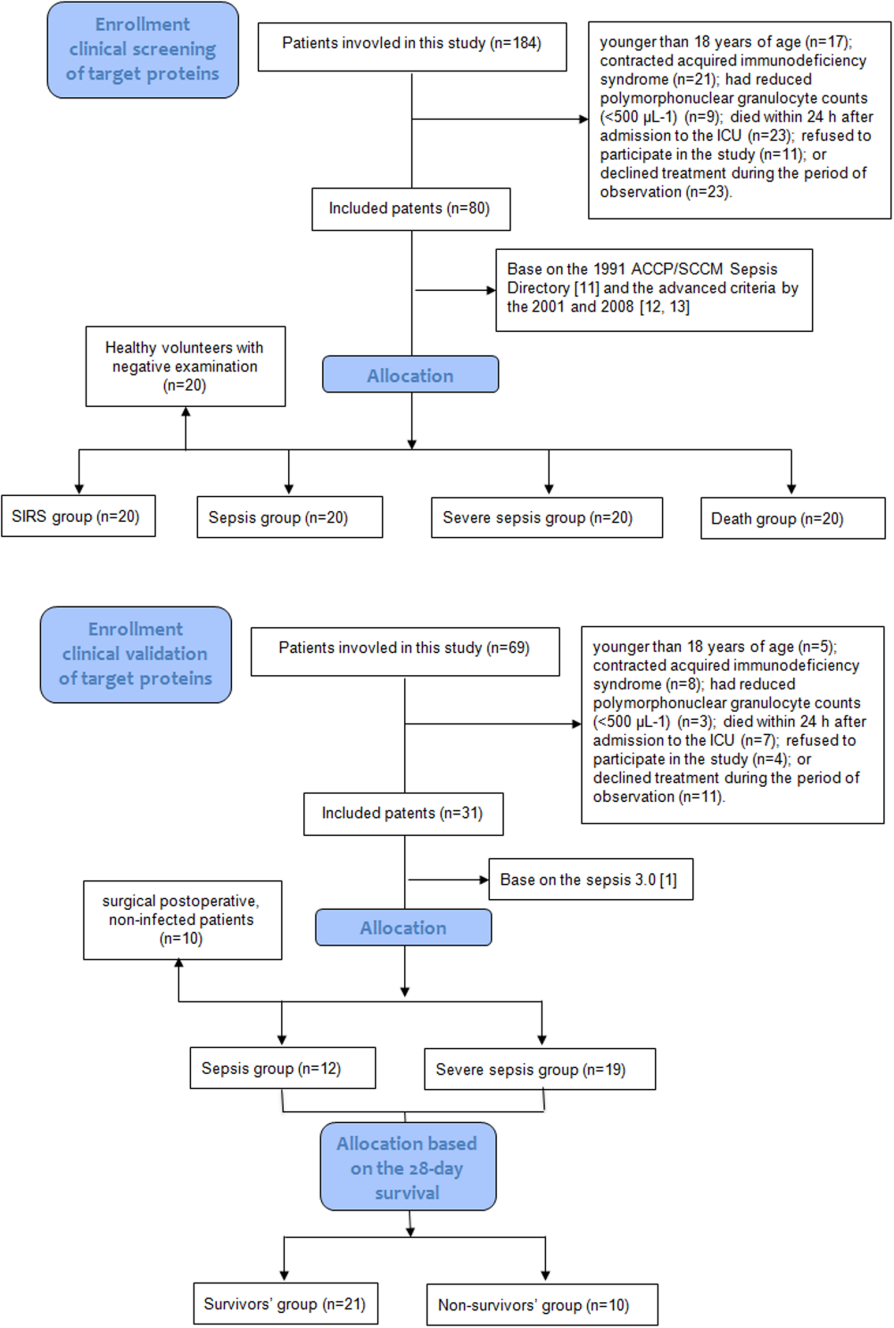
Flowchart illustrating the clinical screening stage and clinical validation stage of the target proteins.

A flowchart of the patients included in this study is shown in Fig. 1. The discovery stage and the verification stage were performed in different groups of patients. Table 1 lists the clinical information for all patients enrolled into this study. The WBC level in the control groups from the two different stages was much lower than that of the sepsis group (*p* < 0.05). Acute Physiology, Age, Chronic Health E valuation II (APACHE II) scores, and Sequential Organ Failure Assessment (SOFA) scores increased markedly according to the disease severity (*p* < 0.05). Statistically, there were no differences in gender, age, pathogens detected, or etiological factors among the different groups from the two stages.

**Table 1:** Demographics of the study subjects in the discovery and verification stages.

Characteristics	CON	Target Screening Stage					Clinical Validation Stage			
		SIRS	Sepsis	Severe sepsis	Death	*P* value	CON	sepsis	septic shock	*P* value
	N = 20	N = 20	N = 20	N = 20	N = 20		N = 10	N = 12	N = 19	
Age (years)	61.5 ± 14.2	57.7 ± 16.8	63.8 ± 17.7	69.6 ± 14.2	59.0 ± 17.4	0.158	64.7 ± 16.1	58.1 ± 12.2	68.9 ± 12.5	0.104
Gender (n, %)						0.456				0.201
Male	11 (55)	12 (60)	11 (55)	15 (75)	15 (75)		6 (60)	11 (91.7)	15 (8.9)	
Female	9 (45)	8 (40)	9 (45)	5 (25)	5 (25)		4 (40)	1 (8.3)	4 (21.1)	
WBC counts (×10^9^/L)	7.9 ± 2.9	16.4 ± 4.2	10.9 ± 3.9	13.4 ± 7.9	13.3 ± 7.6	<0.001	6.6 ± 1.2	11.9 ± 7.9	15.7 ± 7.5	0.005
APACHE II score	—	8 ± 3	13.2 ± 6.5	19.5 ± 6.5	23.4 ± 5.4	<0.001	10 ± 4	17.9 ± 6.7	25.4 ± 6.1	<0.001
SOFA score	—	—	3.9 ± 2.5	8.6 ± 4.1	12.2 ± 3.4	<0.001	—	9.5 ± 3.5	13.0 ± 3.7	0.015
Pathogens detected (n, %)										
Gram-positive bacteria	—	—	6 (30)	7 (35)	8 (40)	0.803	—	7 (58.3)	10 (52.6)	0.756
Gram-negative bacteria	—	—	16 (80)	13 (65)	16 (80)	0.449	—	10 (83.3)	16 (86.2)	0.948
Fungi	—	—	5 (25)	8 (40)	8 (40)	0.517	—	5 (41.7)	7 (36.8)	0.788
Etiological factors (n, %)										
Pulmonary infection	—	—	14 (70)	14 (70)	16 (80)	0.711	—	11 (91.7)	15 (78.9)	0.348
Abdominal infection	—	—	4 (20)	5 (25)	2 (10)	0.459	—	4 (33.3)	6 (31.6)	0.919
Bacteremia	—	—	5 (25)	3 (15)	5 (25)	0.675	—	2 (16.7)	2 (10.5)	0.619
Urinary infection	—	—	4 (20)	6 (30)	4 (20)	0.689	—	4 (33.3)	6 (31.6)	0.919
Trauma/soft tissue infection	—	—	4 (20)	7 (35)	7 (35)	0.49	—	4 (33.3)	11 (57.9)	0.183
Others	—	—	0 (0)	2 (10)	1 (5)	0.349	—	0 (0)	2 (10.5)	0.245

*A patient might have more than one pathogenic bacterial infection. Therefore, the sum of the frequencies (pathogens detected and etiological factors) was higher than effective, and the sum of the percentages could be greater than 100%.

### Global description of serum proteomic characteristics and bioinformatics

**Figure 2:**
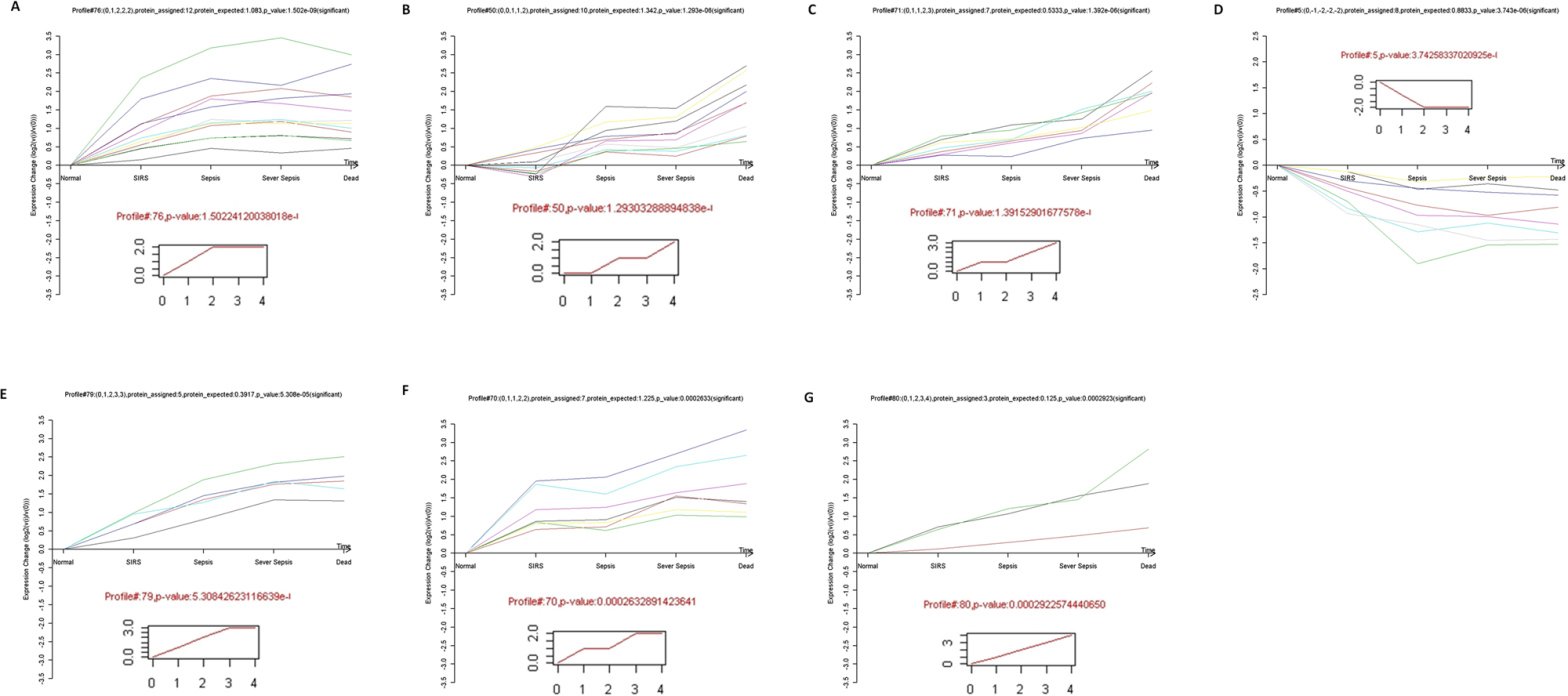
Protein expression patterns analyzed by series test of clusters. The expression patterns of 167 proteins were analyzed, and eight model profiles were used to summarize. Seven protein expression patterns showed significant *p*-values (*p* < 0.05). Inserts represent an identical expression trend for several column molecules. All of the molecules exhibiting this expression trend were displayed in different colored lines above the X-axis. Detailed information is shown in Supplemental Table 2.

**Figure 3:**
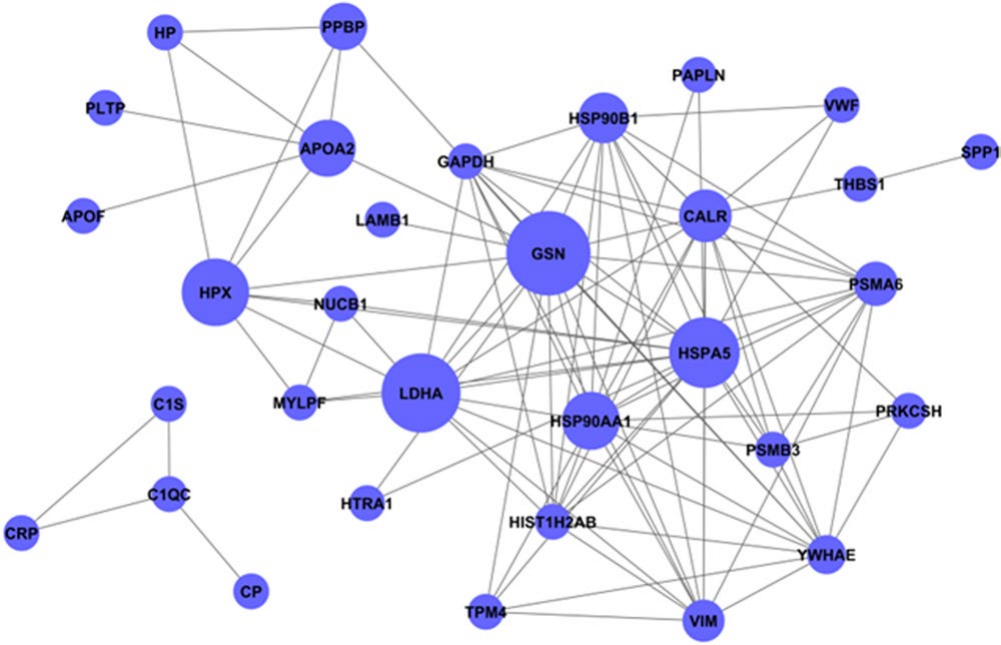
Coexpression network of the proteins. Fifty-two proteins were analyzed and identified based on a protein coexpression network with k-core algorithm. Each circle represented one protein of potential interest. The size of each circle indicated the power of the interrelation among the proteins, and edges between two circles indicated interactions between proteins. The more edges of a given target, the more proteins connect to it and the more central role it plays within the network.

Three technical replicates were analyzed to demonstrate the reproducibility of the experimental results and to perform complementation. A total of 341 proteins were identified, of which 219 proteins were found in all three sets (64.2%). Following the criteria for identifying differentially expressed proteins (fold-change ratio ≥1.2 and *p*-value < 0.05), 192 differentially expressed proteins were selected and built with SI/CON, SE/CON, SS/CON, and DE/CON sequence data based on the fold-change (Supplemental Table 1). Based on cluster analysis of protein expression dynamics, these differentially expressed proteins allocated into 80 expression trend clusters, and seven expression patterns of proteins showed significant *p*-values (*p* < 0.05) (Supplemental Table 2). As shown in Fig. 2, seven of the trend clusters (including 52 proteins) were statistically significant. These 52 proteins were further analyzed and identified by protein coexpression network with k-core algorithm. Detailed information concerning the protein-protein interactions is shown in Fig. 3 and Table 2. Of these proteins, 12 were further selected as potential targets. To ensure the accuracy of the validation, we reevaluated the number of peptides and spectra for these 12 proteins. We found that only three proteins (hemopexin, vimentin, and heat shock protein 90) were eligible by the criteria of a spectrum ≥3 and peptides ≥2. In addition, hemopexin, vimentin, and heat shock protein 90 also had a higher degree of protein-protein interactions, i.e., 8, 11, 15 respectively. HSP90 is a well-established player in the inflammatory response, while hemopexin has fewer protein interactions than vimentin. Therefore, we focused on vimentin as the key target protein for further investigation.

**Table 2:** Information concerning the protein-protein interaction.

Gene symbol	Description	Betweenness centrality	Degree	In-degree	Out-degree
GSN	gelsolin	0.995319495	16	7	9
LDHA	lactate dehydrogenase A	0.773504274	13	8	5
HSPA5	heat shock 70 kDa protein 5 (glucose-regulated protein, 78 kDa)	0.70978836	19	14	5
HPX	hemopexin	0.564102564	8	4	4
HSP90AA1	heat shock protein 90 kDa alpha (cytosolic), class A member 1	0.288970289	15	14	1
APOA2	apolipoprotein A-II	0.273504274	6	3	3
CALR	calreticulin	0.165628816	14	4	10
HSP90B1	heat shock protein 90 kDa beta (Grp94), member 1	0.131440781	13	5	8
PPBP	pro-platelet basic protein (chemokine (C-X-C motif) ligand 7)	0.051282051	4	2	2
PSMA6	proteasome (prosome, macropain) subunit, alpha type, 6	0.029487179	11	1	10
VIM	vimentin	0.020960521	11	5	6
YWHAE	tyrosine 3-monooxygenase/tryptophan 5-monooxygenase activation protein, epsilon polypeptide	0.013105413	13	11	2

Betweenness centrality, the center of the signal; the greater the value, the stronger the intermediate ability of a protein in signal transmission.

Degree, the number of proteins interacting with proteins in the network.

In-degree, the number of proteins upstream of the indicated protein.

Out-degree, the number of downstream proteins of the indicated protein.

### Vimentin expression differentiates sepsis severity and prognosis

**Figure 4:**
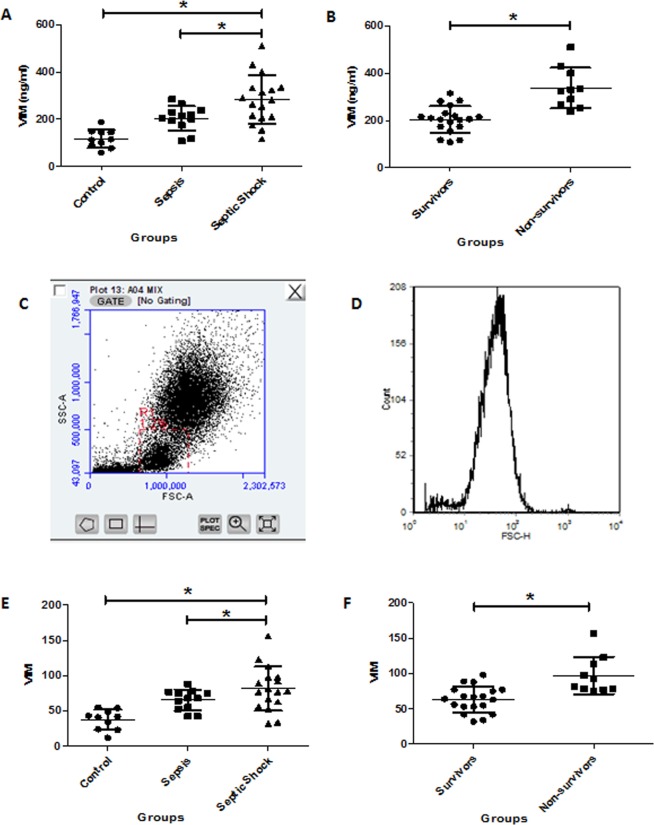
Comparison of serum vimentin concentrations and expression in lymphocytes. Serum vimentin (VIM) concentrations in sepsis patients and vimentin (VIM) expression in lymphocytes isolated from sepsis patients were quantified by ELISA and flow cytometry, respectively, as described in the Materials and Methods. Panel A,B: Comparison of serum vimentin (VIM) concentrations in the different groups by ELISA. Panel C,D: Representative dot plots and histograms of the flow cytometry analysis. Panel E,F: Comparison of vimentin (VIM) expression in lymphocytes isolated from control, sepsis and septic shock patients. *p < 0.05.

Serum vimentin concentrations and its expression in lymphocytes are shown in Fig. 4. The serum concentrations of vimentin in sepsis or septic shock were significantly higher than that in the control group (204.34 ± 52.53 ng/ml or 283.06 ± 102.52 ng/ml vs. 117.36 ± 38.93 ng/ml, respectively, *p* < 0.001). The vimentin expression in lymphocytes showed the same tendency, i.e., the septic shock had the highest, the control group had the lowest, and the sepsis group had moderate level of expression (81.65 ± 29.06 and 37.7 ± 14.25 vs. 65.42 ± 14.65, respectively, *p* < 0.001). The sepsis and septic shock groups were further divided into survivors and nonsurvivors by 28-day survival. This analysis revealed that the nonsurvivor group had higher serum vimentin levels (337.9 ± 86.06 ng/ml vs. 174.43 ± 66.05 ng/ml, *p* < 0.001) and vimentin expression in lymphocytes (97.2 ± 26.39 vs. 54.41 ± 20.89, *p* < 0.001).

### Role of vimentin in mediating survival of lymphocytes

**Figure 5:**
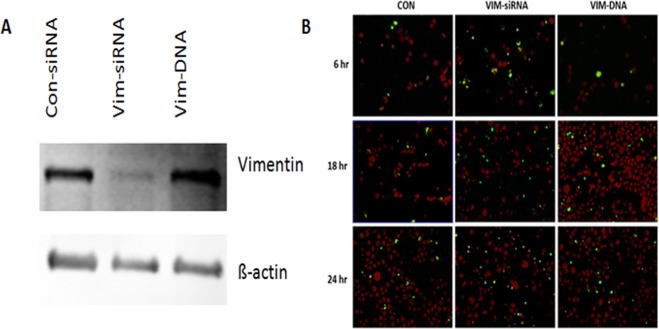
Vimentin expression in Jurkat cells and its effects on apoptosis. Jurkat cells were transfected with control siRNA, vimentin-specific siRNA or a plasmid expressing vimentin as described in the Materials and Methods. Cells were cultured in serum-free DMEM as indicated, followed by TUNEL assay analysis as described in the Materials and Methods. Panel A: Representative immunoblot indicating suppression of vimentin by siRNA (Vim-siRNA) or overexpression of vimentin (Vim-DNA). Panel B: Representative images of TUNEL assays in the absence of LPS. Green: TUNEL-positive staining; Red: cell nuclei.

To further investigate the role of vimentin in the context of sepsis, apoptosis was evaluated in Jurkat cells lacking or overexpressing vimentin. As shown in Fig. 5A, vimentin was either suppressed by vimentin-specific siRNA (Vim-siRNA) or overexpressed by plasmid transfection (Vim-DNA). Following transfection of siRNA or plasmid, the cells were cultured in serum-free DMEM for the indicated time and apoptosis was assessed by TUNEL assay. As shown in Fig. 5B, apoptosis was slightly but not significantly increased in the control Jurkat T cells over 24 h (2.11 ± 0.02% at time zero vs. 3.21 ± 0.90% at 24 h, *p* > 0.05) or cells overexpressing vimentin (Vim-DNA, 3.12 ± 0.22% at time zero vs. 6.62 ± 2.0% at 24 h, *p* > 0.05). In contrast, apoptosis was significantly enhanced in cells lacking vimentin (Vim-siRNA, 3.20 ± 1.02% at time zero vs. 12.16 ± 4.32% at 24 h, *p* < 0.05). Preliminary experiments demonstrated that cells transfected with “control-siRNA” or “pCMV3-untagged negative control vector” expressed the same level of vimentin. Therefore, data for the cells transfected with “pCMV3-untagged negative control vector” were not shown.

**Figure 6:**
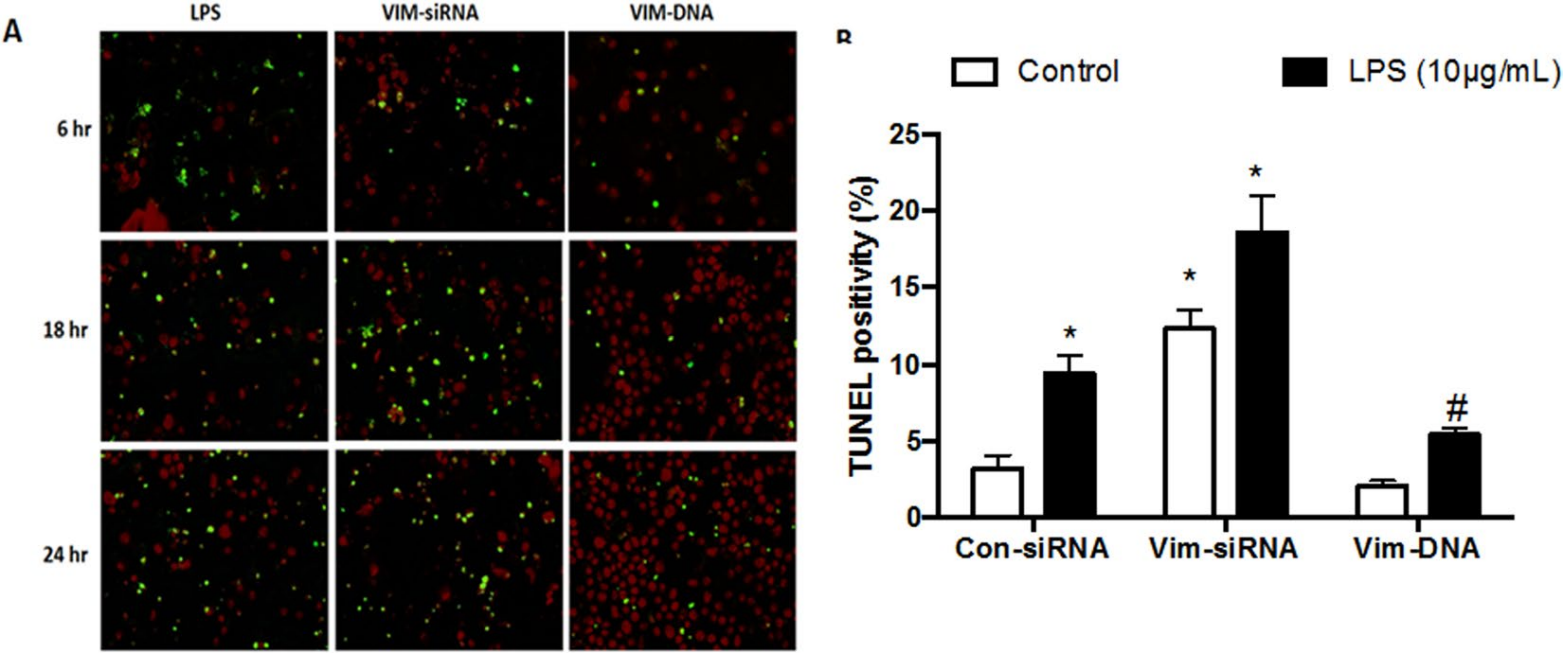
Role of vimentin in modulating Jurkat cell apoptosis in response to LPS. Jurkat cells were transfected with control siRNA, vimentin-specific siRNA or a plasmid expressing vimentin as described in the Materials and Methods. Cells were then cultured for 6, 18 and 24 h in the presence or absence of LPS (10 µg/mL). Panel A: Representative images of TUNEL staining in the presence of LPS. Panel B: Quantitative comparison of TUNEL positivity in cells with or without LPS exposure. *p < 0.05 compared to control cells without LPS; ^#^*p* < 0.05 compared to control cells with LPS. Data shown are from a single representative assessment; the experiments were repeated at least 3 times with similar results.

To further confirm the role of vimentin in modulating lymphocyte apoptosis in the context of sepsis, the cells were exposed to LPS (10 µg/mL) following transfection of vimentin-specific siRNA or plasmid. As shown in Fig. 6, after 6, 18 and 24 h exposure to LPS, nearly 10% of the cells transfected with control siRNA had undergone apoptosis at 24 h (3.21 ± 0.90% of control vs. 9.42 ± 1.24% of LPS-stimulated cells at 24 h, *p* < 0.05), which was significantly increased in the cells lacking vimentin and exposed to LPS (12.16 ± 4.32% vs. 18.63 ± 2.42%, *p* < 0.05, Fig. 6A,B). In contrast, apoptosis was significantly reduced in the cells over-expressing vimentin in the presence of LPS (5.44 ± 1.02%, *p* < 0.05 compared to the LPS alone, Fig. 6A,B).

### Mechanism of apoptosis in the cells lacking vimentin

**Figure 7:**
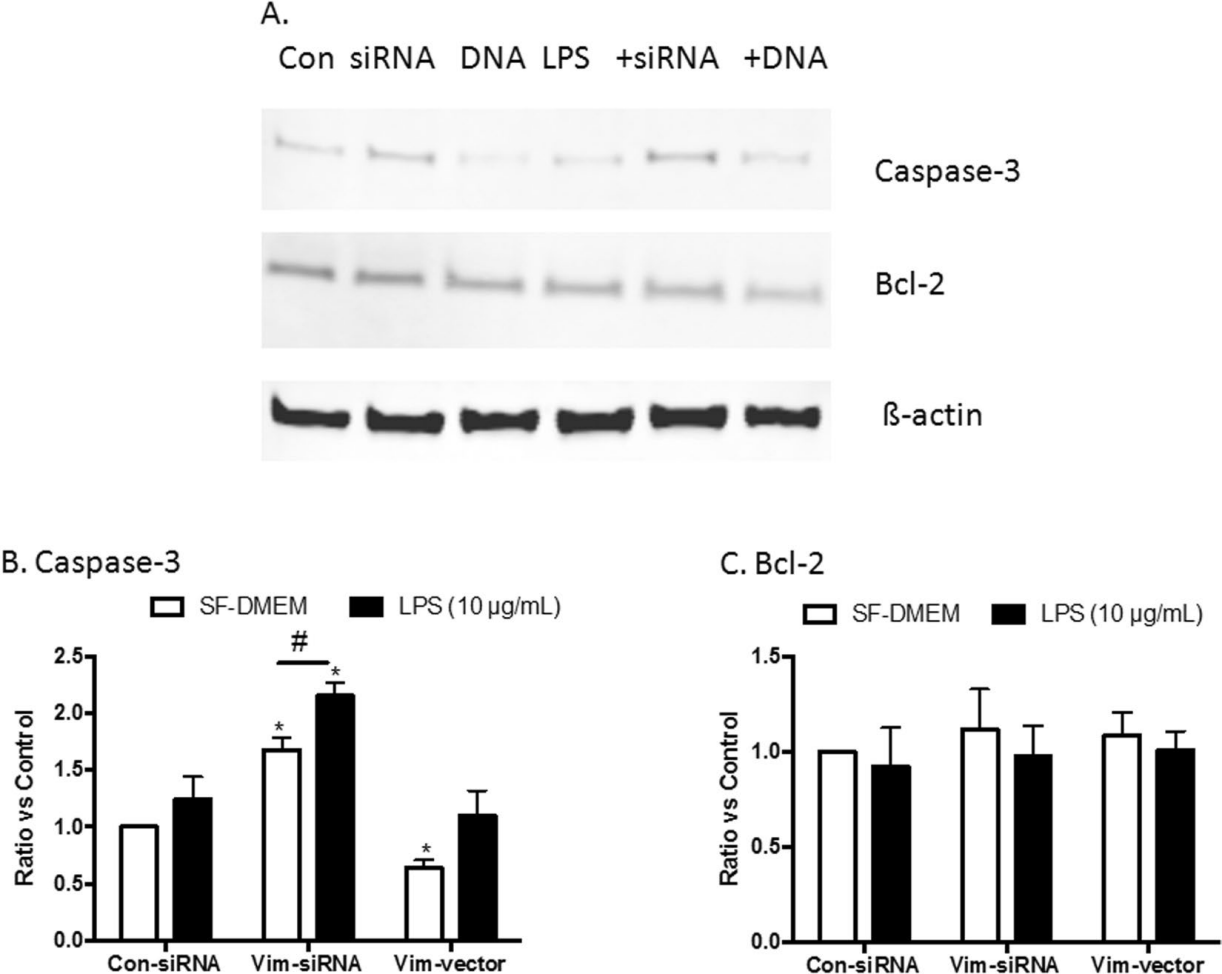
Expression of caspase-3 and Bcl-2 in Jurkat cells lacking vimentin or overexpressing vimentin. Jurkat cells were transfected with control siRNA, vimentin-specific siRNA or a plasmid expressing vimentin as described in the Materials and Methods. Cells were then cultured in serum-free DMEM in the presence or absence of LPS (10 µg/mL) for 24 h. Total cell lysates were analyzed via immunoblotting for caspase-3, Bcl-2 and ß-actin (as a loading control) as described in the Materials and Methods. Panel A: Representative immunoblots. Panel B: Semiquantitative comparison of target proteins with normalization. Vertical axes: protein level expressed as “Ratio versus control”, which was obtained as follows: (1). The densities of target protein versus internal control ß-actin were obtained. (2). The group of cells transfected with Con-siRNA and cultured in SF-DMEM was set as a reference (“Control = 1”), and the ratio of other groups versus this control group were obtained. Horizontal axes: cells transfected with control siRNA (Con-siRNA), vimentin-specific siRNA (Vim-siRNA) or vimentin expressing plasmid (Vim-vector). Open bar: cells were cultured in serum-free DMEM (SF-DMEM); closed bar: cells treated with 10 µg/mL LPS. ^#^*p* < 0.05; *p < 0.05 compared to cells transfected with Con-siRNA and cultured in SF-DMEM. Data represent an average of 3 separate experiments.

To further investigate the potential mechanism of apoptosis in the cells lacking vimentin, the level of pro-apoptotic and anti-apoptotic proteins were assessed by immunoblotting in Jurkat cells following transfection of vimentin-specific siRNA or the plasmid. As shown in Fig. 7, the caspase-3 protein was increased over 60% in the cells lacking vimentin compared with the cells transfected with control siRNA (1.68 ± 0.11-fold increase, *p* < 0.05, Fig. 7A,B). Similarly, in the presence of LPS, caspase-3 was significantly upregulated in the cells lacking vimentin compared with the control cells (1.24 ± 0.20-fold increase of the control cells exposed to LPS vs. 2.25 ± 0.12-fold increase in the cells lacking vimentin plus LPS, *p* < 0.05). In contrast, caspase-3 was reduced nearly 40% in the cells over-expressing vimentin in the absence of LPS (0.64 ± 0.07-fold change, *p* < 0.05, Fig. 7A,B). In the presence of LPS, however, the difference in caspase-3 level between the control cells and vimentin overexpressing cells was not significant (1.10 ± 0.22-fold changes in the cells overexpressing vimentin plus LPS, *p* > 0.05).

Additional apoptosis-associated proteins, including caspase-8, caspase-9, Bim, Bax, and PARP-1, were also assessed. However, none of these proteins was significantly changed in cells lacking vimentin or overexpressing vimentin (data not shown).

### Pro-inflammatory and anti-inflammatory cytokine production

**Figure 8:**
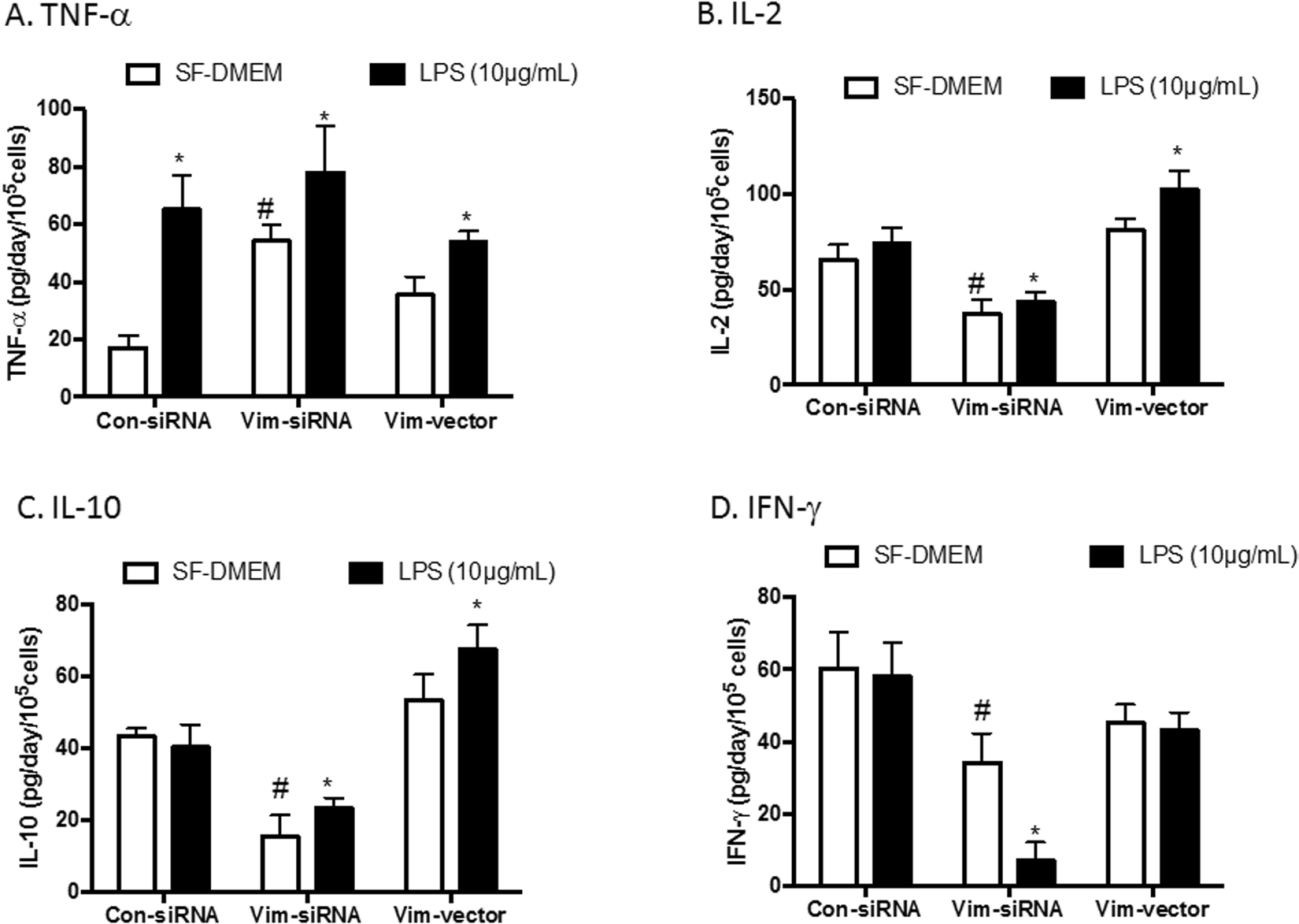
Release of cytokines in the supernatants. Jurkat cells were transfected with control siRNA, vimentin-specific siRNA or a plasmid expressing vimentin as described in the Materials and Methods. Cells were then cultured in serum-free DMEM in the presence or absence of LPS (10 µg/mL) for 24 h. Supernatants were harvested and used for quantification of cytokines by ELISA as described in the Materials and Methods. Panel A: TNF-α. Panel B: IL-2. Panel C: IL-10. Panel D: IFN-γ. Vertical axes: cytokine level expressed as pg/day/10^5^ cells; horizontal axes: cells transfected with control siRNA (Con-siRNA), vimentin-specific siRNA (Vim-siRNA) or the plasmid expressing vimentin (Vim-vector). Data are shown from one representative assessment; experiments were each repeated at least 3 times with similar results. Open bar: cells cultured in the serum-free DMEM (SF-DMEM); closed bar: cells treated with 10 µg/mL LPS. *p < 0.05 compared to cells cultured in SF-DMEM; ^#^*p* < 0.05 compared to cells transfected with Con-siRNA and cultured in SF-DMEM.

Since inflammatory cytokines are involved in sepsis, production of pro-inflammatory and anti-inflammatory cytokines was assessed by ELISA in the current study. As shown in Fig. 8A, in the absence of LPS, TNF-α was significantly increased in Jurkat cells transfected with vimentin-specific siRNA (54.1 ± 5.8 pg/day/10^5^ cells) compared to cells transfected with control siRNA (17.1 ± 4.3 pg/day/10^5^ cells, *p* < 0.05), while TNF-α production in the cells overexpressing vimentin was not significantly altered compared with the control (35.1 ± 6.5 pg/day/10^5^ cells, *p* > 0.05). LPS alone stimulated TNF-α in the cells transfected with control siRNA (65.1 ± 12.3 pg/day/10^5^ cells, *p* < 0.05), but LPS did not further stimulate TNF-α production in the cells transfected with vimentin-specific siRNA (78.1 ± 16.2 pg/day/10^5^ cells, *p* > 0.05) or the cells overexpressing vimentin (54.2 ± 3.6 pg/day/10^5^ cells, *p* > 0.05). However, this treatment did slightly increase TNF-α production (Fig. 8A). In contrast, IL-2, IL-10 and IFN-γ levels were significantly decreased in cells lacking vimentin (IL-2: 37.5 ± 7.3 pg/day/10^5^ cells, Fig. 8B; IL-10: 15.5 ± 5.9 pg/day/10^5^ cells, Fig. 8C; IFN-γ: 34.2 ± 8.1 pg/day/10^5^ cells, Fig. 8D) compared to control cells (IL-2: 65.3 ± 8.3 pg/day/10^5^ cells, Fig. 8B; IL-10: 43.3 ± 2.2 pg/day/10^5^ cells, Fig. 8C; IFN-γ: 60.3 ± 10.1 pg/day/10^5^ cells, Fig. 8D, *p* < 0.05), whereas they were either slightly increased (IL-2: 80.9 ± 10.3 pg/day/10^5^ cells, Fig. 8B and IL-10: 53.3 ± 7.1 pg/day/10^5^ cells, Fig. 8C) or had no significant changes (IFN-γ: 45.3 ± 4.9 pg/day/10^5^ cells, Fig. 8D) in the cells overexpressing vimentin. Furthermore, production of these cytokines was significantly altered in the presence of LPS in the three types of cells (Fig. 8B–D). IL-1ß and IL-12 were not detectable in the current study.

## Discussion

We have identified vimentin as a potential target protein in patients with different stages of sepsis through iTRAQ protein quantification and further verified that vimentin may be used to differentiate between sepsis and septic shock or even predict the prognosis of sepsis. In this context, vimentin expression increased in lymphocytes isolated from sepsis patients. Findings from our *in vitro* experiments with Jurkat cells indicated that vimentin may protect lymphocytes from apoptosis and that suppression of vimentin in lymphocytes may augment the inflammatory response in sepsis through upregulation of inflammatory cytokine release.

Vimentin is a type III intermediate filament protein that is expressed in mesenchymal cells^30^. Intermediate filaments along with tubulin-based microtubules and actin-based microfilaments comprise the cytoskeleton. All intermediate filament proteins are expressed in a highly developmentally regulated fashion, and vimentin, as a main intermediate filament in a variety of cells, is the major cytoskeletal component of mesenchymal cells. Therefore, vimentin is often used as a marker of mesenchymal-derived cells or cells undergoing an epithelial-to-mesenchymal transition (EMT) during both normal development and metastatic progression. Vimentin plays a significant role in supporting and anchoring the position of the organelles in the cytosol. Vimentin is attached to the nucleus, endoplasmic reticulum, and mitochondria, either laterally or terminally^31^. Clinically and traditionally, vimentin has been used as a sarcoma tumor marker to identify mesenchyme^32^. In the current study, screening of proteomics results revealed that serum vimentin concentration increased at different stages of sepsis. In addition, the differential protein interaction network indicated that vimentin had interactions with other proteins, including heat shock protein 90 (HSP90), transmembrane protein 4 (TMP4), and 14-3-3 protein epsilon (YWHAE). These findings suggested that vimentin might be involved in transmembrane signal transduction and could be a potential new target for sepsis diagnosis and prognosis.

Recently, vimentin has been reported to be involved in several inflammatory diseases. Mor-Vaknin *et al*. challenged wild-type and vimentin-knockout mice with *Escherichia coli* intraperitoneally to induce colitis and found that macrophages of vimentin-knockout mice showed a significantly increased capacity to mediate bacterial killing via abundant production of ROS and nitric oxides^33^, suggesting that vimentin impeded bacterial killing and contributed to the pathogenesis of acute colitis. In addition, it has also been reported that serum citrullinated and MMP-degraded vimentin levels were increased in inflammatory bowel disease and could therefore serve as a biomarker with high diagnostic power^34^. Furthermore, autoantibodies against vimentin or citrullinated vimentin have been found to be involved in the pathogenesis of a variety of autoimmune diseases, including systemic lupus erythematosus (SLE)^35^, rheumatoid arthritis^36^, sarcoidosis^37^, and idiopathic pulmonary fibrosis^38^. However, evidence of vimentin involvement in sepsis has not previously been reported. In this study, we found that serum vimentin concentrations increased during sepsis, suggesting that vimentin might be cleaved and released from the cytosol into the circulation. This finding indicates that vimentin degradation may be increased in cells following the increased expression of vimentin in lymphocytes in sepsis. Therefore, soluble vimentin in the circulation could be a biomarker for the diagnosis and prognosis of sepsis.

Vimentin has been suggested to play a role in the pathogenesis of inflammatory diseases. In this regard, Dos Santos, *et al*. used a LPS-induced acute lung injury mouse model to demonstrate that lung inflammation and fibrosis were attenuated in the lungs of vimentin-knockout mice compared to wild-type controls; this effect may have been due to decreased activation of the NLRP3 inflammasome^39^. Thiagarajan *et al*. reported that vimentin could function as an endogenous, activating ligand for Dectin-1, the nontoll pattern recognition receptor (PRR), on monocytes and that activation of monocytes contributed to the chronic inflammation associated with atherosclerosis^40^. Toda *et al*. reported that vimentin bound to phosphorylated p38 MAPK and mediated CCL2 production in mast cells, which could be a key mechanism of allergic inflammation^40^. Li *et al*. reported that culture of endothelial cells with serum from a murine model of sepsis induced by cecal ligation and puncture (CLP) resulted in phosphorylation of vimentin filaments, which was strongly associated with VE-cadherin and led to increased permeability of endothelial cells^41^. Here, we used Jurkat cells to explore the role of vimentin in modulating the inflammatory reaction of lymphocytes in response to LPS stimulation. We found that an inflammatory mediator, TNF-α, was significantly increased, whereas the anti-inflammatory cytokine IL-10 was decreased in cells lacking vimentin, suggesting that vimentin may be involved in regulating inflammation in response to endotoxin exposure in lymphocytes.

In sepsis, in addition to the “cytokine storm”, apoptotic cell death presents in a variety of cell types, including lymphocytes. In this regard, after the acute phase of sepsis, apoptosis of lymphoid cells and suppression of lymphocyte responses is believed to contribute to the development of infectious complications often seen in septic patients^42–46^. Vimentin has also been reported to be involved in regulating apoptosis. Hsu *et al*. reported that overexpression of peptidylarginine deiminase type 2 (PADI2) induced apoptosis in PMA plus ionomycin-activated Jurkat cells through increases in vimentin citrullination^14^. Moisan *et al*. reported that intracellular vimentin was cleaved and expressed on the cell surface during spontaneous or agent-induced human neutrophil apoptosis^15^. Byun *et al*. demonstrated that vimentin was rapidly proteolyzed by caspase-3, -6 and -7 into similar-sized fragments during apoptosis in response to a variety of stimuli, and suggested that cleavage of vimentin promoted apoptosis by disrupting the cytoplasmic network^18^. Lee *et al*. reported that palmitate and LPS had synergistic effects on the activation of caspase-3 and vimentin expression and cleavage^47^. These researchers reported that caspase-3 was activated by palmitate but not by LPS; however, vimentin expression was stimulated by LPS. Furthermore, a combination of palmitate and LPS stimulation resulted in a significant increase in cleaved vimentin and secreted vimentin levels in culture supernatants^47^. In this study, while we did not examine whether LPS stimulated vimentin expression or there was a direct effect of LPS on caspase-3 activation, we found that caspase-3 levels were increased in Jurkat cells following suppression of vimentin by siRNA, although it was not significantly increased by LPS. In contrast, caspase-3 levels were decreased in cells overexpressing vimentin, suggesting that vimentin may regulate caspase-3. This finding may be correlated with vimentin-associated apoptosis in Jurkat cells.

## Conclusions

The current study demonstrated that serum vimentin concentration was significantly increased in patients with sepsis, which may result from the increased expression and degradation of vimentin in lymphocytes isolated from septic patients. *In vitro* studies indicated that suppression of vimentin in Jurkat cells by siRNA resulted in augmentation of cellular apoptosis and pro-inflammatory cytokine release, whereas overexpression of vimentin protected cells from apoptosis. These findings suggest that vimentin could serve as a biomarker for the diagnosis and prognosis of sepsis or septic shock and that vimentin is involved in lymphocyte apoptosis and inflammatory cytokine release in response to endotoxin exposure.

### Ethical Approval and Consent to participate

Study Protocol of clinical screening stage was approved by the Ethics Committee of the Chinese PLA General Hospital (project No. 20111013-007) and was registered with the Clinical Trials Register (NCT 01493466). The Study Protocol of clinical validation stage was approved by the Ethics Committee of the Peking Union Medical College Hospital (project No.ZS1080) and was registered with the Clinical Trials Register (NCT 03253146). Participants or their family members were fully informed of the study and signed consent forms were obtained.

## Supplementary information

Supplemental Table1

Supplemental Table2

## Data Availability

All the dada can be provided for all the reader. Contact with the corresponding author Prof. Dawei Liu or Prof. Lixin Xie.
