# Supplementary material for: Role of vimentin in modulating immune cell apoptosis and inflammatory responses in sepsis
Source: Sci Rep. 2019 Apr 5;9:5747. doi: 10.1038/s41598-019-42287-7 (PMC6451033; doi:10.1038/s41598-019-42287-7)
Supplement: Supplementary file 1 — Supplemental Table1 [file 41598_2019_42287_MOESM1_ESM.pdf]

Role of vimentin in modulating immune cell apoptosis and inflammatory response in sepsis

Longxiang Su1,*, Pan Pan2,*, Peng Yan3, Yun Long1, Xiang Zhou1, Xiaoting Wang1, Ruo Zhou4, Bo Wen4, Lixin Xie3,#, Dawei Liu1,#1

1 Department of Critical Care Medicine, Peking Union Medical College Hospital, Peking Union Medical College, Chinese Academy of Medical Sciences, 1 Shuaifuyuan, Dongcheng District, Beijing 100730, China
2 Department of Critical Care Medicine, Beijing Tiantan Hospital, Capital Medical University, Tiantan Xili the 6th, Dongcheng District, Beijing 100050, China
3 Shenzhen Proteome Engineering Laboratory, BGI Shenzhen, Shenzhen, China
4 Department of Respiratory and Critical Care Medicine, Chinese PLA General Hospital, 28thFuxing Rd, Haidian District, Beijing 100853, China

* Longxiang Su and Pan Pan contributed equally to this study.

#Corresponding author:
Lixin Xie, M.D.
Director and Professor
Department of Respiratory and Critical Care Medicine, Chinese PLA General Hospital, 28thFuxing Rd, Haidian District, Beijing 100853, China
Tel/Fax:+86 10 66876432
E-mail:xielx301@126.com
OR
Dawei Liu, M.D.
Director and Professor
Department of Critical Care Medicine, Peking Union Medical College Hospital, Peking Union Medical College, Chinese Academy of Medical Sciences, 1 Shuaifuyuan, Dongcheng District, Beijing 100730, China
Tel/fax: +86 10 69152305
E-mail: dwliu98@163.com

Accession	proteins	CON	SIRS	Sepsis/Severe sepsis	Septic shock	Dead	model number	p_value	
IPI00022418	FN1	0	-0.25177	-0.923466153	-1.120573127	-1.55008	1	0.00715937	
IPI00922213	FN1	0	-0.209557	-0.899691248	-1.181208588	-1.613846	1	0.00715937	
IPI00030739	APOM	0	-0.163069	-0.37517848	-0.680774426	-0.588805	2	0.00075689	
IPI00291262	CLU	0	-0.358396	-0.461179281	-0.647084213	-0.650765	2	0.00075689	
IPI00022295	PF4V1	0	-0.715747	-1.281549892	-1.79285535	-1.744879	2	0.00075689	
IPI00007221	SERPINA5	0	-0.74932	-1.142740171	-1.443253008	-1.678072	2	0.00075689	
IPI00021855	APOC1	0	-0.271027	-0.573374526	-0.68466915	-0.524732	3	0.02051787	
IPI00022446	PF4	0	-0.591679	-0.90560763	-1.286289758	-1.033276	3	0.02051787	
IPI00029061	SEPP1	0	-0.228562	-0.415037499	-0.585283065	-0.375178	3	0.02051787	
IPI00305461	ITIH2	0	-0.617612	-1.445196984	-1.529237923	-1.836867	4	0.01687063	
IPI00022331	LCAT	0	-0.445727	-0.809002774	-0.918386235	-1.203306	4	0.01687063	
IPI00032179	SERPINC1	0	-0.37592	-0.578214166	-0.665695953	-1.061891	4	0.01687063	
IPI00021854	APOA2	0	-0.121236	-0.459781321	-0.355768059	-0.474047	5	3.74E-06	*
IPI00022731	APOC4	0	-0.439002	-0.771463059	-0.965691949	-0.80653	5	3.74E-06	*
IPI00021364	CFP	0	-0.703101	-1.903231131	-1.537296067	-1.525568	5	3.74E-06	*
IPI00299503	GPLD1	0	-0.295527	-0.44148348	-0.516015148	-0.57628	5	3.74E-06	*
IPI00026314	GSN	0	-0.835385	-1.283131579	-1.112588939	-1.304316	5	3.74E-06	*
IPI00218732	PON1	0	-0.498081	-0.965445691	-0.992526753	-1.137504	5	3.74E-06	*
IPI00022445	PPBP	0	-0.119909	-0.316145743	-0.229792983	-0.224863	5	3.74E-06	*
IPI00296099	THBS1	0	-0.933321	-1.147523792	-1.449957483	-1.431356	5	3.74E-06	*
IPI00186903	APOL1	0	-0.789381	-1.647544769	-1.758587454	-0.958533	6	0.8319173	
IPI00029739	CFH	0	-0.158337	-0.545638948	-0.244887058	-0.39835	7	0.83472143	
IPI00304273	APOA4	0	-1.049166	-1.321735724	-0.773433761	-0.551885	8	0.10860981	
IPI00011264	CFHR1	0	-0.590722	-0.962363931	-0.586884814	-0.651224	8	0.10860981	
IPI00003351	ECM1	0	-0.78799	-1.211842879	-0.701327258	-0.536716	8	0.10860981	
IPI00384280	PCYOX1	0	-0.249749	-0.503348735	-0.279768422	-0.212881	8	0.10860981	
IPI00292950	SERPIND1	0	-0.611015	-1.03703073	-0.730313886	-0.468844	8	0.10860981	
IPI00019568	F2	0	-0.175045	-0.257815244	-0.349742699	-0.756454	10	0.25935648	
IPI00027410	GP5	0	-1.18248	-0.938850337	-1.651836229	-1.45242	11	0.73982507	
IPI00328609	SERPINA4	0	-1.170993	-1.417920008	-1.947853143	-1.257815	12	0.6971134	
IPI00023019	SHBG	0	-1.596299	-1.335711912	-2.582475716	-1.718088	12	0.6971134	

IPI00064667	CNDP1	0	-0.321928	-0.175471374	-0.246104011	-0.684968	13	0.31425282	
IPI00022937	F5	0	-0.283724	-0.201633862	-0.287275273	-0.71604	13	0.31425282	
IPI00298971	VTN	0	-0.286881	-0.510624408	-0.486113662	-1.183116	13	0.31425282	
IPI00012269	MMRN1	0	-0.710173	-0.42115596	-0.659924559	-0.812293	14	0.62221268	
IPI00299738	PCOLCE	0	-0.385891	-0.216196155	-0.229382834	-0.273018	14	0.62221268	
IPI00163207	PGLYRP2	0	-1.411788	-1.737975127	-1.76525184	-1.455229	14	0.62221268	
IPI00022229	APOB	0	-0.360645	-0.738407569	-0.66781645	0.274622	15	0.87835724	
IPI00844511	MAN1A1	0	-0.532068	-0.301782724	-0.263835681	-0.065343	15	0.87835724	
IPI00745872	ALB	0	-0.301002	-0.261029271	-0.037499367	-0.197446	16	0.49129918	
IPI00022391	APCS	0	-0.567545	-0.646162657	-0.188611921	-0.4868	16	0.49129918	
IPI00293925	FCN3	0	-0.453122	-0.356895018	-0.058431953	-0.321928	16	0.49129918	
IPI00410297	HPSE	0	-0.345964	-0.728573602	-0.011018478	-0.527738	16	0.49129918	
IPI00020091	ORM2	0	-0.604704	-0.367743806	-0.179723351	-0.574667	16	0.49129918	
IPI00032220	AGT	0	-1.826464	-1.487486349	-0.742437446	-0.045443	17	0.19189238	
IPI00006662	APOD	0	-0.426175	-0.532400086	-0.174619473	0.03357	17	0.19189238	
IPI00020986	LUM	0	-0.168214	-0.364012054	0.214240225	0.216474	17	0.19189238	
IPI00299307	MASP1	0	-0.313246	-0.411426246	-0.09017595	-0.041243	17	0.19189238	
IPI00553177	SERPINA1	0	-0.892974	-0.577569818	0.024247547	0.044439	17	0.19189238	
IPI00296537	FBLN1	0	-0.332851	-0.283329168	-0.012211084	0.233617	18	0.53077191	
IPI00023673	LGALS3BP	0	-0.207476	-0.154021453	0.150934826	0.3046	18	0.53077191	
IPI00000075	TGFB1	0	-0.266037	0.007956723	-0.601221086	-0.793272	19	0.82469821	
IPI00029260	CD14	0	-0.766383	0.118204885	-1.098733954	-0.30529	21	0.93401257	
IPI00383629	IGHV1-24	0	-0.363638	-0.074962057	-0.578214166	-0.111477	21	0.93401257	
IPI00022426	AMBP	0	-0.221155	-0.113700499	-0.030924573	-0.421515	22	0.98088691	
IPI00029658	EFEMP1	0	-0.518703	0.035539755	0.547228873	0.606767	26	0.48930319	
IPI00218803	FBLN1	0	-0.180572	0.103146927	0.293948399	0.263612	26	0.48930319	
IPI00021841	APOA1	0	-0.035624	-0.248939575	-0.400173566	-0.479575	29	0.3627103	
IPI00783987	C3	0	-0.004322	-0.306067767	-0.335330799	-0.474047	31	0.34664896	
IPI00292530	ITIH1	0	-0.065572	-0.619647767	-0.803640358	-1.315276	31	0.34664896	
IPI00006173	CETP	0	0.5152603	-0.66205355	-0.882838656	-0.001922	33	0.95379256	
IPI00021856	APOC2	0	0.0019249	-0.730313886	-0.021953441	0.218151	35	0.96774876	
IPI00298828	APOH	0	0.0588937	-0.841168043	0.215077299	0.357529	35	0.96774876	
IPI00292532	CAMP	0	-0.009347	-0.271425677	0.148800661	0.03357	35	0.96774876	
IPI01014270	APOB	0	-0.209141	-0.834306702	-0.267036336	0.605303	36	0.91993764	

IPI00889723	C4A	0	0.0795797	0.597277823	-1.139251187	-0.178024	39	0.96774876	
IPI00896380	IGHM	0	0.0028883	0.125530882	-0.452771126	-0.041243	39	0.96774876	
IPI00009890	SERPINE2	0	0.2083943	0.292475209	-0.32998465	0.12658	39	0.96774876	
IPI00329555	F7	0	-0.021953	0.160040413	-0.19870368	-0.953327	40	0.86548599	
IPI00025052	FCN2	0	-0.195348	-0.197236356	0.125793214	-0.556797	40	0.86548599	
IPI00022432	TTR	0	0.0573917	0.060898825	0.268237479	-0.339517	40	0.86548599	
IPI00784154	HSPD1	0	-0.014831	0.30638295	0.015957574	2.836501	41	0.62221268	
IPI00020996	IGFALS	0	0.0355398	-0.204558102	-0.391034457	0.523532	41	0.62221268	
IPI00009792	IGHV1OR15-1	0	-0.056584	0.382074667	0.28865194	2.383329	41	0.62221268	
IPI00022246	AZU1	0	0.2150773	0.305788392	1.446148032	0.291592	42	0.99450313	
IPI00294713	MASP2	0	-0.193246	-0.218677856	0.511827796	0.321327	43	0.8388406	
IPI00003590	QSOX1	0	0.081105	0.12973393	0.515260334	0.884523	44	0.22132549	
IPI00027827	SOD3	0	0.0614005	-0.16650122	0.328555647	0.787534	44	0.22132549	
IPI00021578	CFHR4	0	0.047915	0.457989645	0.25785003	-0.24894	45	0.92642209	
IPI00479708	IGHM	0	0.0553914	0.543719519	-0.276198864	0.003852	46	0.99450313	
IPI00021842	APOE	0	-0.058432	0.182240324	-0.308011314	0.282205	47	0.77818406	
IPI00654755	HBB	0	0.1844246	1.298081353	0.60823228	1.522841	47	0.77818406	
IPI00386879	IGHA1	0	-0.154021	0.552861658	0.099020517	0.5132	47	0.77818406	
IPI00022392	C1QA	0	0.1103967	1.119249181	0.635614336	0.284546	48	0.87835724	
IPI00785084	IGHV4-31	0	-0.1137	1.687334826	0.823677227	0.367732	48	0.87835724	
IPI00930442	IGHG4	0	-0.106125	0.69276579	0.695878402	0.577767	49	0.50772631	
IPI00418153	IGHM	0	0.0877334	0.992326072	0.644607789	0.5828	49	0.50772631	
IPI00020599	CALR	0	0.0969617	0.946193557	1.19759996	2.173536	50	1.29E-06	
IPI00022200	COL6A3	0	-0.16092	0.367111404	0.249822294	0.793357	50	1.29E-06	*
IPI00017601	CP	0	-0.224452	0.385839859	0.465938398	0.650635	50	1.29E-06	*
IPI00382470	HSP90AA1	0	0.4435297	0.783389931	0.859875776	2.005782	50	1.29E-06	*
IPI00154742	IGLC2	0	-0.070389	0.422752465	0.379882835	0.814768	50	1.29E-06	*
IPI00217966	LDHA	0	-0.311116	0.66352617	0.694321256	1.703689	50	1.29E-06	*
IPI00026154	PRKCSH	0	0.4887006	1.177881725	1.302819272	2.576332	50	1.29E-06	*
IPI00028004	PSMB3	0	0.0923402	0.572751417	0.479954975	1.052895	50	1.29E-06	*
IPI00296083	SFTPB	0	-0.236748	1.600920074	1.54793177	2.695878	50	1.29E-06	*
IPI00418471	VIM	0	0.3352138	0.703689439	0.880090537	1.694321	50	1.29E-06	*
IPI00006543	CFHR5	0	-0.106572	0.119249181	0.499537844	0.140826	51	0.01063214	
IPI00387022	LOC100128009	0	0.1031469	0.487351704	1.171368418	0.817731	51	0.01063214	

IPI00903112	LTF	0	0.0977334	0.462952481	0.763660461	0.186886	51	0.01063214	
IPI00019038	LYZ	0	-0.13137	0.271136204	0.620009448	0.233334	51	0.01063214	
IPI00027933	PSMB10	0	0.1546772	1.181149439	2.300448367	1.300448	51	0.01063214	
IPI00295400	WARS	0	-0.198494	1.051399152	1.260151897	0.631511	51	0.01063214	
IPI00007244	MPO	0	0.1767941	0.779257316	1.425333315	1.100567	52	0.3627103	
IPI00032293	CST3	0	0.0626556	0.191820917	0.822401071	1.064917	53	0.061383	
IPI00294615	FBLN5	0	0	0.350154647	0.502259911	0.913216	53	0.061383	
IPI00021817	PROC	0	0.5070359	-0.078154366	-0.229792983	-0.41684	55	0.84019083	
IPI00022431	AHSG	0	0.8050725	0.051897579	0.235031127	0.042457	58	0.75705173	
IPI00291867	CFI	0	0.6719295	0.356605546	0.2544041	0.111435	58	0.75705173	
IPI00022371	HRG	0	0.438959	-0.213295433	-0.154885602	-0.188612	58	0.75705173	
IPI00654888	KLKB1	0	0.4073636	-0.226097446	-0.006716916	-0.108803	58	0.75705173	
IPI00032328	KNG1	0	0.5842413	-0.004800986	0.297490205	-0.012926	60	0.96550656	
IPI00294004	PROS1	0	0.4357031	0.09439237	0.412474985	0.137648	60	0.96550656	
IPI00887154	C4B	0	0.6311385	-0.099407428	1.138176939	0.703689	61	0.68016255	
IPI00479116	CPN2	0	0.3529158	0.057391663	0.256126037	0.385212	61	0.68016255	
IPI00291866	SERPING1	0	0.3971948	-0.064882851	0.581360264	0.387726	61	0.68016255	
IPI00021857	APOC3	0	0.3189256	-0.015782998	0.099020517	0.541618	62	0.53579324	
IPI00026199	GPX3	0	0.117683	-0.085424656	0.207005867	0.486004	62	0.53579324	
IPI00014572	SPARC	0	0.3965616	0.529072743	0.034061836	-1.088594	63	0.81407052	
IPI00423462	IGHA1	0	0.3346072	0.578484937	-0.295135249	-0.326921	64	0.87835724	
IPI00025426	PZP	0	1.6635262	1.504987124	0.684240574	0.429213	64	0.87835724	
IPI00854743	LOC652113	0	0.9105018	0.468930507	0.151201819	0.558517	65	0.98088691	
IPI00021727	C4BPA	0	0.3529158	0.407363571	0.429860943	-0.069473	66	0.58978058	
IPI00025862	C4BPB	0	0.3727045	0.296899301	0.370215978	0.005782	66	0.58978058	
IPI00019580	PLG	0	0.2020186	0.286890765	0.314732594	-0.194087	66	0.58978058	
IPI00032291	C5	0	1.3340009	1.154142004	1.180059378	0.926865	67	0.11545966	
IPI00879709	C6	0	1.243546	1.024247546	0.980891177	0.92139	67	0.11545966	
IPI00296608	C7	0	1.3051941	1.011587974	1.0489096	0.896111	67	0.11545966	
IPI00294395	C8B	0	0.8961108	0.822826331	0.557100725	0.421464	67	0.11545966	
IPI00011261	C8G	0	1.0449352	0.87743771	0.906890596	0.698217	67	0.11545966	
IPI00896419	ITIH4	0	1.1616533	1.57132159	1.452056689	1.520769	67	0.11545966	
IPI00010491	RAB27B	0	1.3352138	1.340075442	1.055891201	1.547932	67	0.11545966	
IPI00975939	SAA2-SAA4	0	0.7784322	0.569893179	0.621488376	0.515948	67	0.11545966	

IPI00478003	A2M	0	0.240134	0.277533975	0.309955453	0.684241	68	0.00391498	
IPI00011062	CPS1	0	0.8916428	1.605302949	0.671929501	3.782562	68	0.00391498	
IPI00021885	FGA	0	2.6958784	2.326745111	1.556393349	3.260728	68	0.00391498	
IPI00298497	FGB	0	2.8503071	2.265344567	1.513199832	3.380822	68	0.00391498	
IPI00021891	FGG	0	3.0750134	2.062906752	1.084160448	3.762844	68	0.00391498	
IPI00453473	HIST1H4D	0	1.0019249	1.611167571	1.702123845	3.936037	68	0.00391498	
IPI00003935	HIST2H2BE	0	0.8219759	1.198703359	1.206450878	3.41632	68	0.00391498	
IPI00296165	C1R	0	0.9592244	1.092340172	1.459311399	1.017417	69	0.46561508	
IPI00019591	CFB	0	0.6119023	0.387097109	0.716275736	0.51939	69	0.46561508	
IPI00022420	RBP4	0	0.4331037	0.271136204	1.10159814	0.513886	69	0.46561508	
IPI00022895	A1BG	0	0.8624965	0.909146569	1.514573173	1.401635	70	0.00026329	*
IPI00022394	C1QC	0	0.643105	0.72102405	1.552156356	1.337643	70	0.00026329	*
IPI00010295	CPN1	0	0.8365013	0.623709616	1.036525876	0.988504	70	0.00026329	*
IPI00297284	IGFBP2	0	1.9620321	2.066931008	2.695878402	3.341293	70	0.00026329	*
IPI00032311	LBP	0	1.8677522	1.609699179	2.346175641	2.655929	70	0.00026329	*
IPI00002352	MYLPF	0	1.1844246	1.249250591	1.639354798	1.887189	70	0.00026329	*
IPI00294705	PAPLN	0	0.8067539	0.827939254	1.184424571	1.115077	70	0.00026329	*
IPI00219018	GAPDH	0	0.6966576	1.097476151	1.251538767	2.559225	71	1.39E-06	*
IPI00026272	HIST1H2AB	0	0.3723932	0.666576266	0.942029931	2.234465	71	1.39E-06	*
IPI00027230	HSP90B1	0	0.7941905	0.957355663	1.433103684	1.952694	71	1.39E-06	*
IPI00031821	ITM2B	0	0.2749133	0.243830672	0.735763849	0.954557	71	1.39E-06	*
IPI00643034	PLTP	0	0.472596	0.701341684	1.521459558	2.007715	71	1.39E-06	*
IPI00029623	PSMA6	0	0.2986727	0.615581735	0.862496476	1.965784	71	1.39E-06	*
IPI00023014	VWF	0	0.6214884	0.716275736	1.028165254	1.487352	71	1.39E-06	*
IPI00465378	APOA5	0	0.5563933	0.762028453	0.239566125	0.008199	72	0.62194858	
IPI00022395	C9	0	0.8651219	1.299264375	0.708396442	0.346176	72	0.62194858	
IPI00011252	C8A	0	0.7652943	0.926865295	0.678841548	0.463947	73	0.73310963	
IPI00026944	NID1	0	0.5556863	0.938799843	0.642354164	0.568466	73	0.73310963	
IPI00784842	IGHV4-31	0	0.1806043	1.292180752	0.504987124	0.987551	74	0.51805901	
IPI00178926	IGJ	0	0.1260556	0.582079992	0.317726358	0.590021	74	0.51805901	
IPI00296176	F9	0	1.1088401	1.477944251	1.342512427	0.930527	75	0.08862746	
IPI00387168	PCSK9	0	0.3971948	1.114035244	0.903288359	0.481969	75	0.08862746	
IPI00006146	SAA2	0	1.12134	2.398461943	2.655929022	1.72261	75	0.08862746	
IPI00552578	SAA2	0	0.7036894	1.785875195	1.94712044	1.253831	75	0.08862746	

IPI00020019	ADIPOQ	0	0.1509348	0.461296305	0.341902795	0.465274	76	1.50E-09	*
IPI00017696	C1S	0	0.5606428	1.077041036	1.193194781	0.905989	76	1.50E-09	*
IPI00022389	CRP	0	2.358454	3.180059379	3.452714702	3	76	1.50E-09	*
IPI00431645	HP	0	1.1297339	1.579921884	1.813499443	1.943416	76	1.50E-09	*
IPI00003176	HTRA1	0	0.7450029	1.150934826	1.241270432	1.007715	76	1.50E-09	*
IPI00028413	ITIH3	0	0.9105018	1.795859283	1.677302673	1.475268	76	1.50E-09	*
IPI00328703	OAF	0	0.6495028	1.129733929	1.134477041	1.12658	76	1.50E-09	*
IPI00022429	ORM1	0	0.5367263	1.242407755	1.17679413	1.22095	76	1.50E-09	*
IPI00007199	SERPINA10	0	0.4546905	0.747422866	0.804232464	0.72738	76	1.50E-09	*
IPI00550991	SERPINA3	0	1.1140352	1.874789753	2.079071571	1.852042	76	1.50E-09	*
IPI00296777	SPARCL1	0	0.4593114	0.739372092	0.816884065	0.670398	76	1.50E-09	*
IPI00010779	TPM4	0	1.8008774	2.35107444	2.171368418	2.740175	76	1.50E-09	*
IPI00021439	ACTB	0	0.5145732	1.552156356	1.736965594	2.426625	77	0.00355502	
IPI00555812	GC	0	0.6943213	1.553567305	1.717856771	2.514573	77	0.00355502	
IPI00021263	YWHAZ	0	1.2845459	1.717856771	1.915935735	2.965784	77	0.00355502	
IPI00477992	C1QB	0	0.5936447	1.071977166	1.46461056	1.083141	78	0.29553813	
IPI00299435	APOF	0	0.3153308	0.812654526	1.346175641	1.311148	79	5.31E-05	*
IPI00013976	LAMB1	0	0.6943213	1.352915787	1.76121314	1.852042	79	5.31E-05	*
IPI00295542	NUCB1	0	0.9856447	1.888968688	2.317127109	2.509088	79	5.31E-05	*
IPI00021000	SPP1	0	0.691212	1.457989644	1.819427754	1.984693	79	5.31E-05	*
IPI00031008	TNC	0	0.9573557	1.269976015	1.839940352	1.637857	79	5.31E-05	*
IPI00022488	HPX	0	0.7186479	1.066931008	1.550746785	1.888969	80	5.31E-05	*
IPI00003362	HSPA5	0	0.117683	0.291592016	0.475936324	0.692766	80	5.31E-05	*
IPI00000816	YWHAE	0	0.6446078	1.209784021	1.457989644	2.822826	80	0.00029226	*
