# Supplementary material for: Role of vimentin in modulating immune cell apoptosis and inflammatory responses in sepsis
Source: Sci Rep. 2019 Apr 5;9:5747. doi: 10.1038/s41598-019-42287-7 (PMC6451033; doi:10.1038/s41598-019-42287-7)
Supplement: Supplementary file 2 — Supplemental Table2 [file 41598_2019_42287_MOESM2_ESM.pdf]

Role of vimentin in modulating immune cell apoptosis and inflammatory response in sepsis

Longxiang Su1,*, Pan Pan2,*, Peng Yan3, Yun Long1, Xiang Zhou1, Xiaoting Wang1, Ruo Zhou4, Bo Wen4, Lixin Xie3,#, Dawei Liu1,#1

1 Department of Critical Care Medicine, Peking Union Medical College Hospital, Peking Union Medical College, Chinese Academy of Medical Sciences, 1 Shuaifuyuan, Dongcheng District, Beijing 100730, China
2 Department of Critical Care Medicine, Beijing Tiantan Hospital, Capital Medical University, Tiantan Xili the 6th, Dongcheng District, Beijing 100050, China
3 Shenzhen Proteome Engineering Laboratory, BGI Shenzhen, Shenzhen, China
4 Department of Respiratory and Critical Care Medicine, Chinese PLA General Hospital, 28thFuxing Rd, Haidian District, Beijing 100853, China

* Longxiang Su and Pan Pan contributed equally to this study.

#Corresponding author:
Lixin Xie, M.D.
Director and Professor
Department of Respiratory and Critical Care Medicine, Chinese PLA General Hospital, 28thFuxing Rd, Haidian District, Beijing 100853, China
Tel/Fax:+86 10 66876432
E-mail:xielx301@126.com
OR
Dawei Liu, M.D.
Director and Professor
Department of Critical Care Medicine, Peking Union Medical College Hospital, Peking Union Medical College, Chinese Academy of Medical Sciences, 1 Shuaifuyuan, Dongcheng District, Beijing 100730, China
Tel/fax: +86 10 69152305
E-mail: dwliu98@163.com

Accession	genesymbol	Normal	SIRS	Sepsis	Sever Sepsis	Dead	modelnumber	Figure2
IPI00020019	ADIPOQ	0	0.150934826	0.461296305	0.341902795	0.465274327	76	A
IPI00017696	C1S	0	0.560642822	1.077041036	1.193194781	0.905989193	76	A
IPI00022389	CRP	0	2.358453971	3.180059379	3.452714702	3	76	A
IPI00431645	HP	0	1.129733929	1.579921884	1.813499443	1.943416472	76	A
IPI00003176	HTRA1	0	0.745002914	1.150934826	1.241270432	1.007714965	76	A
IPI00028413	ITIH3	0	0.910501849	1.795859283	1.677302673	1.475267636	76	A
IPI00328703	OAF	0	0.649502753	1.129733929	1.134477041	1.126580497	76	A
IPI00022429	ORM1	0	0.536726315	1.242407755	1.17679413	1.220950447	76	A
IPI00007199	SERPINA10	0	0.454690547	0.747422866	0.804232464	0.727379545	76	A
IPI00550991	SERPINA3	0	1.114035244	1.874789753	2.079071571	1.852042119	76	A
IPI00296777	SPARCL1	0	0.459311399	0.739372092	0.816884065	0.670397977	76	A
IPI00010779	TPM4	0	1.800877358	2.35107444	2.171368418	2.740175151	76	A
IPI00020599	CALR	0	0.09696173	0.946193557	1.19759996	2.173536255	50	B
IPI00022200	COL6A3	0	-0.160920188	0.367111404	0.249822294	0.793356776	50	B
IPI00017601	CP	0	-0.224451944	0.385839859	0.465938398	0.650634723	50	B
IPI00382470	HSP90AA1	0	0.44352971	0.783389931	0.859875776	2.005782353	50	B
IPI00154742	IGLC2	0	-0.070389328	0.422752465	0.379882835	0.814767746	50	B
IPI00217966	LDHA	0	-0.311115554	0.66352617	0.694321256	1.703689439	50	B
IPI00026154	PRKCSH	0	0.488700648	1.177881725	1.302819272	2.576332196	50	B
IPI00028004	PSMB3	0	0.092340172	0.572751417	0.479954975	1.052894949	50	B
IPI00296083	SFTPB	0	-0.236747715	1.600920074	1.54793177	2.695878402	50	B
IPI00418471	VIM	0	0.335213786	0.703689439	0.880090537	1.694321257	50	B
IPI00219018	GAPDH	0	0.696657606	1.097476151	1.251538767	2.559224939	71	C
IPI00026272	HIST1H2AB	0	0.372393162	0.666576266	0.942029931	2.234465254	71	C
IPI00027230	HSP90B1	0	0.794190463	0.957355663	1.433103684	1.952694285	71	C
IPI00031821	ITM2B	0	0.274913275	0.243830672	0.735763849	0.954557029	71	C
IPI00643034	PLTP	0	0.472595977	0.701341684	1.521459558	2.007714965	71	C
IPI00029623	PSMA6	0	0.298672742	0.615581735	0.862496476	1.965784285	71	C
IPI00023014	VWF	0	0.621488376	0.716275736	1.028165254	1.487351704	71	C
IPI00021854	APOA2	0	-0.121236486	-0.459781321	-0.355768059	-0.474046599	5	D
IPI00022731	APOC4	0	-0.439002491	-0.771463059	-0.965691949	-0.806530289	5	D
IPI00021364	CFP	0	-0.7031007	-1.903231131	-1.537296067	-1.52556779	5	D

IPI00299503	GPLD1	0	-0.295527126	-0.44148348	-0.516015148	-0.576280257	5	D
IPI00026314	GSN	0	-0.835385151	-1.283131579	-1.112588939	-1.304316332	5	D
IPI00218732	PON1	0	-0.498080629	-0.965445691	-0.992526753	-1.137503522	5	D
IPI00022445	PPBP	0	-0.119909463	-0.316145743	-0.229792983	-0.224863495	5	D
IPI00296099	THBS1	0	-0.933320837	-1.147523792	-1.449957483	-1.431355521	5	D
IPI00299435	APOF	0	0.31533085	0.812654526	1.346175641	1.311148256	79	E
IPI00013976	LAMB1	0	0.694321256	1.352915787	1.76121314	1.852042119	79	E
IPI00295542	NUCB1	0	0.985644707	1.888968688	2.317127109	2.509087634	79	E
IPI00021000	SPP1	0	0.691211999	1.457989644	1.819427754	1.984692747	79	E
IPI00031008	TNC	0	0.957355663	1.269976015	1.839940352	1.637857449	79	E
IPI00022895	A1BG	0	0.862496476	0.909146569	1.514573173	1.401634795	70	F
IPI00022394	C1QC	0	0.643104982	0.72102405	1.552156356	1.337642565	70	F
IPI00010295	CPN1	0	0.836501268	0.623709616	1.036525876	0.988504361	70	F
IPI00297284	IGFBP2	0	1.96203215	2.066931008	2.695878402	3.34129342	70	F
IPI00032311	LBP	0	1.867752202	1.609699179	2.346175641	2.655929022	70	F
IPI00002352	MYLPF	0	1.184424571	1.249250591	1.639354798	1.887188681	70	F
IPI00294705	PAPLN	0	0.806753924	0.827939254	1.184424571	1.115076524	70	F
IPI00022488	HPX	0	0.71864794	1.066931008	1.550746785	1.888968688	80	G
IPI00003362	HSPA5	0	0.11768302	0.291592016	0.475936324	0.69276579	80	G
IPI00000816	YWHAE	0	0.644607789	1.209784021	1.457989644	2.822826331	80	G
